# Risk Factors for Serious Suicide Attempts: Difference Between Older and Younger Attempters in the Emergency Department

**DOI:** 10.3389/fpsyt.2020.607811

**Published:** 2021-01-08

**Authors:** Dong Wook Kim, Seo Eun Cho, Jae Myeong Kang, Soo Kyun Woo, Seung-Gul Kang, Byeong Kil Yeon, Seong-Jin Cho

**Affiliations:** ^1^College of Medicine, Gachon University, Incheon, South Korea; ^2^Department of Psychiatry, Gil Medical Center, Gachon University College of Medicine, Incheon, South Korea; ^3^Department of Psychiatry, Gyeonggi Provincial Medical Center Suwon Hospital, Suwon, South Korea

**Keywords:** lethality, old age suicide, serious suicide attempt, risk factor, risk rescue rating scale

## Abstract

**Objective:** Suicide attempts of the older adults are known to be more serious than that of the younger adults. Despite its major social impact in South Korea, the behavioral mechanism of serious suicide attempt (SSA) in old people remains to be elucidated. Thus, we investigated the risk factors for SSA in older and younger suicide attempters in the emergency department.

**Methods:** Demographic data, clinical information, and the level of seriousness of suicide with Risk Rescue Rating Scale were compared between older (age ≥65) and younger (age <65) adults who visited the emergency department for a suicide attempt. Regression analyses were performed to identify the risk factors for SSA in these two groups.

**Results:** Among 370 patients, 37 were older adults (10%; aged 74.41 ± 6.78), more likely to have another medical disease (*p* < 0.001), and a higher suicide completion rate (16.2 vs. 5.4%, *p* = 0.023). In the younger group, old age (*B* = 0.090, *p* < 0.001), male sex (*B* = −0.038, *p* = 0.019), and impression of schizophrenia (*B* = 0.074, *p* = 0.027) were associated with a higher risk-rescue ratio and interpersonal stress condition was associated with a lower risk-rescue ratio (*B* = −0.045, *p* = 0.006). In the older group, however, no variables were included significant in the regression model for the Risk Rescue Rating Scale.

**Conclusions:** Demographic and clinical factors such as old age, male sex, interpersonal stress, and impression of schizophrenia were associated with lethality in the younger suicide attempters. However, no factors were associated with SSA in the older adult group. Different mechanisms may underly the lethality in old age suicide.

## Introduction

Suicide is one of the leading causes of death worldwide (1). Many risk factors and consequences constitute the complexity of suicidal behaviors. Suicide is recognized as a critical health issue throughout the lifespan. The epidemiologic studies have promisingly reported that the global age-standardized mortality rate of suicide decreased, although the number of suicidal deaths increased during the past two decades (1). However, suicide rates in South Korea are still 2.4-folds higher than those for other Organization for Economic Co-operation and Development (OECD) countries (2), ranking the fourth most common cause of all death in 2017 (3).

The outcome of suicide attempts varies from non-lethal suicidal behavior to suicidal completion with death. It is important to measure the lethality of the suicide attempt and prevent serious suicide attempts (SSA), defined as suicide attempt leading to a fatal outcome including death or hospitalization (4, 5) because the SSA cause both individual and social burden (4, 6). Many factors are involved in SSA or suicide lethality, including medical severity (7, 8), previous attempts (9), suicidal intent and method (7, 10), male sex (10), previous history and severity of psychiatric diseases (11–13), alcohol use (14), and age (15). A more comprehensive evaluation of SSA may include contextual information based on methods and circumstances of the suicide attempt because the lethality of the suicide attempt is associated with multiple variables.

Old age is known to be associate with SSA (15). A suicide attempt among the elderly is more serious, with a ~3-fold completion rate than that among non-elderly (16). Thus, studies have investigated risk factors for SSA, particularly in old age. An earlier literature review showed that major psychiatric illnesses, including depressive and psychotic disorders, were associated with SSA in older suicide attempters (17). More recent studies have reported that factors such as physical disease (18), functional disability (19), and social isolation (20) are associated with SSA or completion of suicide among older adults. Because the suicide rate of the older adult in South Korea has been the highest among the OECD countries for 13 consecutive years from 2005 to 2017, except for Lithuania that joined in May 2017 (2), it is necessary to investigate the risk factors associated with SSA in a Korean sample of older people. However, few studies have investigated risk factors for SSA in older adults compared to the younger age groups except for one recent study (21). Comparing the risk factors for SSA between older and younger groups would show information critical for old suicide attempters.

This study compared the characteristics of sociodemographic and clinical information of older and younger suicide attempters. Additionally, we investigated the hypothesis that risk factors for SSA in older adults would differ from those in the younger adults.

## Methods and Materials

### Participants

This retrospective, single-center study was conducted from April 2019 to June 2020 using data from medical records in Gachon University Gil Medical Center. Of the 296,157 eligible patients who visited the emergency department from August 2012 to December 2015, 1,498 patients who were confirmed to have attempted suicide were selected. Selected patients were categorized as “suicide attempt” in the intentionality of injury by an emergency department doctor among “suicide attempt,” “unintentional trauma,” and “violence/homicide.” Of the 1,498 patients, data from 346 participants who agreed to interview with a psychiatrist were used. Participants aged 65 or above were classified as the older adult group. The age of 65 was set because of the consensus statement on the psychiatry of the elderly produced by World Health Organization and World Psychiatric Association (22) and the subsequent publication mostly used 65 years as the cut-off age for depression in the elderly (23–25). All participants were interviewed by an emergency medical doctor and a psychiatrist. A patient with a suicide attempt is first screened by an emergency department doctor and then consulted by a psychiatrist to confirm if they attempted suicide, intervened with supportive psychotherapy, and set a treatment plan. Within a relatively short time, a semi-structured interview is required asking details of the suicide attempt and the risk factors. The lethality of the suicide attempt was routinely measured with the Risk Rescue Rating Scale (RRRS). Data of 24 suicide victims during the study period were extracted by reviewing the whole data of death on arrival during the study period to avoid excluding data of suicide completion (death), and RRRS were retrospectively measured with chart review. There were no multiple admissions of one patient that might lead to duplication of data.

### Assessments

All interviews were conducted in the emergency room. Demographic information, including age, sex, years of education, occupation, another medical condition, living with family, marital status, and religion, were gathered. Suicide attempt and clinical information, including method of suicide attempt, precipitating factor, current psychiatric impression, alcohol intoxication, suicidal note, past psychiatric history, previous suicide attempt, and family history of suicide and psychiatric disease were also gathered. The information details were gathered using a semi-structured form from all participants during the interview with a psychiatrist. Besides, we reviewed the medical records of all interviewed participants to find whether they expired after admission to the ward.

According to the Structured Clinical Interview for Diagnostic and Statistical Manual of Mental disorders-IV Axis I disorders (26), mental status and current psychiatric impressions were examined. The RRRS was administered to assess the lethality of the suicide attempt (27). This scale consists of 10 items: five items describe risk factors (the method used, impaired consciousness, toxicity, reversibility, and treatment required) and five describe rescue factors (location, the person initiating a rescue, probability of discovery, accessibility for rescue, and delay until discovery). Higher risk scores (ranging from 1 to 5) indicate more lethal suicide attempts, and higher rescue scores (ranging from 1 to 5) means less serious and more rescuable suicide attempts. The risk/rescue ratio is determined using the formula [Risk score/(Risk score + Rescue score)] × 100. Risk, rescue, and risk/rescue ratio scores were analyzed as continuous variables as there is no cut-off score of RRRS to define a fatal suicide attempt or SSA.

The institutional review board of Gachon University Gil Medical Center approved this study. Because of a retrospective study design, the need for informed consent was waived, and the period of investigation was from April 2019 to June 2020 (approval No. GCIRB2019-126).

### Statistical Analysis

Descriptive statistics were used to analyze socio-demographic, clinical, and suicidal data and RRRS scores. Group comparisons of data were performed using the Mann-Whitney U test for continuous variables and chi-square or Fisher's exact test for categorical variables. Spearman correlation analysis was used to analyze the correlations among the RRRS, socio-demographic, clinical, and suicidal information. The effects of independent demographic, clinical, and suicidal information on RRRS were assessed using multiple stepwise regression analysis after performing correlation analysis. A *p-*value of less than 0.05 (*p* < 0.05) was considered significant. Statistical analyses were performed using the Statistical Package for the Social Sciences (SPSS, version 23, Chicago, IL, USA).

## Results

### Socio-Demographic Characteristics

A total of 370 participants comprised 37 (10.0%) older (74.41 ± 6.78) and 333 (90.0%) younger (37.23 ± 13.37) suicide attempters. Table 1 presents the socio-demographic variables. The years of education of older people were significantly shorter than those of the younger people (7.07 ± 5.47 vs. 11.54 ± 2.88, *p* < 0.001). A significantly higher proportion of old people was unemployed (*p* < 0.001) and had an additional medical condition (*p* < 0.001). No significant differences were found in sex (*p* = 0.342) and cohabitation with family (*p* = 0.770) but marital status (*p* < 0.001) and religion differed between the two groups (*p* = 0.022).

**Table 1:** Demographic and clinical information of younger and older suicide attempters.

**Variable**	**Younger** ** attempters** ** (*n =* 333)**	**Older** ** attempters** ** (*n* = 37)**	**Total** ** (*n* = 370)**	***p*-value**
Age, years (mean ± SD)	37.23 ± 13.37	74.41 ± 6.78	41.00 ± 17.03	** <0.001^†^**
Education, years (mean ± SD)	11.54 ± 2.88	7.07 ± 5.47	11.12 ± 3.45	** <0.001^†^**
Sex, female (*n* [%])	198 (59.5%)	19 (51.4%)	217 (58.6%)	0.342
Occupation (yes; *n* [%])	142 (43.7%)	2 (5.6%)	144 (39.9%)	** <0.001^‡^**
Another medical condition (yes; *n* [%])	75 (22.9%)	26 (70.3%)	101 (27.7%)	** <0.001^‡^**
Living with family *(yes; *n* [%])*	260 (79.0%)	30 (81.1%)	290 (79.2%)	0.770^‡^
Marriage status (*n* [%])				** <0.001^‡^**
Unmarried	132 (40.1%)	0 (0.0%)	132 (36.2%)	
Married	135 (41.0%)	20 (55.6%)	155 (42.5%)	
Widowed	38 (11.6%)	3 (8.3%)	41 (11.2%)	
Separated	11 (3.3%)	12 (33.3%)	23 (6.3%)	
Divorced	13 (4.0%)	1 (2.8%)	14 (3.8%)	
Religion (yes; *n* [%])	76 (25.3%)	15 (45.5%)	91 (27.3%)	**0.022^‡^**

† *Mann-Whitney U-test*;

‡ *Chi-square test or Fisher's exact test*.

*Bold type indicates values significant at p < 0.05*.

### Suicide Attempt and Clinical Information

Table 2 presents information on suicide attempts and clinical characteristics. There were no significant differences in the method of suicide attempt between the two groups (χ^2^ = 6.41, *p* = 0.492), while there was a significant difference in the precipitating factor for suicide attempts (χ^2^ = 47.26, *p* = 0.002). In the younger group, interpersonal problems accounted for 43.1% of suicide attempts, while in the older group, physical illness was the major reason accounting for 37.8% of suicide attempts. Younger attempters showed a higher number of previous suicide attempts than older attempters (χ^2^ = 12.83, *p* = 0.002). There were no differences in the current psychiatric diagnosis, alcohol intoxication, suicide note, past psychiatric history, and family history of suicide attempts and psychiatric diseases. Older attempters showed a higher suicide completion rate than younger attempters (χ^2^ = 6.42, *p* = 0.023).

**Table 2:** Information on suicide attempts in younger and older groups.

**Variable**	**Younger** ** attempters** ** (*n =* 333)**	**Older** ** attempters** ** (*n* = 37)**	**Total** ** (*n* = 370)**	**χ^**2**^, *p*-value**
Method of suicide attempt (*n* [%])				6.41, 0.492
Drug ingestion	179 (54.4%)	24 (64.9%)	203 (55.5%)	
Hanging	42 (12.8%)	3 (8.1%)	45 (12.3%)	
CO intoxication	31 (9.4%)	1 (2.7%)	32 (8.7%)	
Wrist cutting	53 (16.1%)	6 (16.2%)	59 (16.1%)	
Fall down	12 (3.6%)	0 (0.0%)	12 (3.3%)	
Stab injury	7 (2.1%)	2 (5.4%)	9 (2.5%)	
Neck laceration	4 (1.2%)	1 (2.7%)	5 (1.4%)	
Jumping into a car	1 (0.3%)	0 (0.0%)	1 (0.3%)	
Precipitating factor for suicide attempt (*n* [%])				**47.26, 0.002**
Interpersonal problems	143 (43.1%)	8 (21.6%)	151 (40.9%)	
Economic problems	73 (22.0%)	4 (10.8%)	77 (20.9%)	
Physical illness	19 (5.7%)	14 (37.8%)	33 (8.9%)	
Work stresses	20 (6.0%)	0 (0.0%)	20 (5.4%)	
Others^†^	9 (2.7%)	1 (2.7%)	10 (2.7%)	
None	10 (3.0%)	2 (5.4%)	12 (3.3%)	
Unknown	58 (17.5%)	8 (21.6%)	66 (17.9%)	
Current psychiatric impression (*n* [%])				0.98, 0.986
Major depressive disorder	215 (77.6%)	25 (83.3%)	240 (78.2%)	
Schizophrenia	17 (6.1%)	1 (3.3%)	18 (5.9%)	
Bipolar disorder	14 (5.1%)	1 (3.3%)	15 (4.9%)	
Anxiety disorder	1 (0.4%)	0 (0.0%)	1 (0.3%)	
Adjustment disorder	7 (2.5%)	1 (3.3%)	8 (2.6%)	
Conversion disorder	1 (0.4%)	0 (0.0%)	1 (0.3%)	
None	22 (7.9%)	2 (6.7%)	24 (7.8%)	
Alcohol intoxication (yes; *n* [%])	168 (52.3%)	14 (40.0%)	182 (51.1%)	1.92, 0.166
Suicide note (yes; *n* [%])	18 (5.6%)	1 (2.8%)	19 (5.3%)	0.51, 0.473
Past psychiatric Hx (yes; *n* [%])	120 (42.9%)	8 (29.6%)	128 (41.7%)	1.77, 0.183
Previous suicide attempt (yes; *n* [%])	132 (39.6%)	5 (13.5%)	137 (37.0%)	**12.83, 0.002**
Family Hx of suicide attempt (yes; *n* [%])	18 (6.9%)	0 (0.0%)	18 (6.4%)	1.63, 0.201
Family Hx of psychiatric diseases (death; *n*, [%])	34 (13.0%)	0 (0.0%)	34 (12.0%)	3.326, 0.071
Suicide completion (death; *n*, [%])	18 (5.4%)	6 (16.2%)	24 (6.5%)	**6.42, 0.023**

*Chi-square test or Fisher's exact was used*.

† *Others: death of an acquaintance, abortion/pregnancy*.

*Bold type indicates values significant at p < 0.05*.

*Hx, history*.

Table 3 presents the risk score, rescue score, and risk-rescue ratio score of the younger and older groups comparable between groups.

**Table 3:** Scores of risk rescue rating scale of the suicide attempters.

**Variable**	**Younger attempters** ** (*n =* 333)**	**Older attetmpters** ** (*n* = 37)**	**Z, *p*-value**
Risk score	2.64 ± 1.09	2.78 ± 1.08	−0.86, 0.392
Agent used	1.29 ± 0.53	1.16 ± 0.37	−1.22, 0.223
Impaired consciousness	1.47 ± 0.61	1.43 ± 0.65	−0.58, 0.560
Lesion toxicity	1.97 ± 0.69	2.11 ± 0.70	−1.16, 0.249
Reversibility	1.84 ± 0.65	1.92 ± 0.68	−0.64, 0.523
Treatment required	2.26 ± 0.59	2.30 ± 0.62	−0.45, 0.651
Rescue score	3.92 ± 1.04	4.03 ± 0.83	−0.27, 0.788
Location	2.80 ± 0.49	2.84 ± 0.50	−0.67, 0.504
Person initiating rescue	2.71 ± 0.57	2.81 ± 0.52	−1.29, 0.196
Probability of discovery by a rescuer	2.37 ± 0.65	2.59 ± 0.50	−1.87, 0.062
Accessibility to rescue	1.92 ± 0.79	1.78 ± 0.82	−1.01, 0.312
Delay until discovery	2.57 ± 0.71	2.46 ± 0.77	−0.27, 0.788
Risk-rescue ratio score	39.98 ± 0.14	40.23 ± 0.13	−0.13, 0.894

*Data are mean ± standard deviation values, and the Mann-Whitney U-test was used*.

### Factors Associated With Risk of Suicide Attempt

In multivariate regression analyses, variables such as age, sex, marital status, alcohol intoxication, interpersonal stress, economic stress, schizophrenia, and bipolar disorder were set as independent variables with the stepwise method as they showed significant correlation with risk, rescue, and risk-rescue ratio scores (data not presented). Risk, rescue, and risk-rescue ratio scores were used as dependent variables in multivariate linear regression analyses (Table 4).

**Table 4:** Factors associated with serious suicide attempts in younger and older groups.

**Independent variable**	**Risk score**				**Rescue score**				**Risk-rescue ratio score**			
	***B***	**SE**	**β**	***p***	***B***	**SE**	**β**	***p***	***B***	**SE**	**β**	***p***
**In total participants**												
Age	0.547	0.141	0.220	<0.001					0.061	0.018	0.195	0.001
Sex (female)	−0.299	0.125	−0.136	0.017					−0.039	0.016	−0.141	0.012
Married												
Alcohol intoxication					0.257	0.118	0.126	0.031				
Interpersonal stress					0.283	0.129	0.136	0.029	−0.044	0.016	−0.157	0.005
Economic stress					−0.395	0.158	−0.157	0.013				
r/o Schizophrenia					−0.661	0.252	−0.152	0.009	0.070	0.032	0.119	0.033
r/o Bipolar disorder												
	*F* = 11.427 *p* < 0.001				*F* = 8.333, *p* < 0.001				*F* = 8.874, *p* < 0.001			
**In younger attempters**												
Age	0.705	0.163	0.254	<0.001	−0.387	0.158	−0.146	0.015	0.090	0.020	0.256	<0.001
Sex (female)	−0.316	0.130	−0.143	0.016					−0.038	0.016	−0.136	0.019
Married												
Alcohol intoxication					0.326	0.127	0.157	0.011				
Interpersonal stress					0.301	0.138	0.143	0.030	−0.045	0.016	−0.162	0.006
Economic stress					−0.366	0.168	−0.146	0.030				
r/o Schizophrenia					−0.709	0.262	−0.164	0.007	0.074	0.033	0.128	0.027
r/o Bipolar disorder												
	*F* = 13.373 *p* < 0.001				*F* = 8.118, *p* < 0.001				*F* = 10.470, *p* < 0.001			
**In older attempters**	No significant linear regression models were found.											

*Multivariate logistic linear regression analyses with the stepwise method were used in each group, and final models were presented*.

*Dependent variables were risk score, rescue score, and risk-rescue ratio score, respectively (log-transformed); independent variables were age (log-transformed), sex (male = 1 and female = 2), married (yes/no), alcohol intoxication (yes/no), interpersonal stress (yes/no), economic stress (yes/no), r/o schizophrenia (yes/no), and r/o bipolar disorder (yes/no)*.

*SE, standard error*.

In the whole sample, the risk score was associated with age (*B* = 0.547, *p* < 0.001) and male sex (*B* = −0.299, *p* = 0.017), and rescue score was associated with alcohol intoxication (*B* = 0.257, *p* = 0.031), interpersonal stress (*B* = 0.283, *p* = 0.029), economic stress (*B* = −0.395, *p* = 0.013), and schizophrenia (*B* = −0.661, *p* = 0.009). Risk-rescue ratio was associated with age (*B* = 0.061, *p* = 0.001), male sex (*B* = −0.039, *p* = 0.012), interpersonal stress (*B* = −0.044, *p* = 0.005), and schizophrenia (*B* = 0.070, *p* = 0.033).

In the younger group, risk score was associated with older age (*B* = 0.705, *p* < 0.001) and male sex (*B* = −0.316, *p* = 0.016). Rescue score was associated with age (*B* = −0.387, *p* = 0.015), alcohol intoxication (*B* = 0.326, *p* = 0.011), interpersonal stress (*B* = 0.301, *p* = 0.030), economic stress (*B* = −0.366, *p* = 0.030), and schizophrenia (*B* = −0.709, *p* = 0.007). Risk-rescue ratio was associated with age (*B* = 0.090, *p* < 0.001), male sex (*B* = −0.038, *p* = 0.019), interpersonal stress (*B* = −0.045, *p* = 0.006), and schizophrenia (*B* = 0.074, *p* = 0.027).

In the older group, however, no variables were included in the final regression model with the stepwise method. The result of *post-hoc* power analysis, that addresses the small sample size in the older group, is presented in Supplementary Figure 1.

## Discussion

The present study aimed to identify the differences in the demographic and clinical information and risk factors for SSA between the younger and older suicide attempters. Differences were found in the educational attainment, medical condition, marital status, religion, precipitating factors for suicide attempt, previous suicide attempt, and suicide completion rate. Factors such as age, sex, alcohol intoxication, interpersonal stress, economic stress, and impression of schizophrenia were associated with SSA in the younger group. In contrast, no factors were found to be associated with SSA in the older group.

Concerning demographic and clinical factors, older suicide attempters were more likely to be less educated, unemployed, and had additional medical conditions than younger attempters. Due to poverty after the Korean War, lack of emphasis on education, and scarcity of resources, the older population in Korea is less educated than the younger population (28). Increased unemployment among the old adults is as expected, considering their mean age (74.41 ± 6.78) and retirement. An economic condition, such as diminished wealth and unemployment, is known to increase suicidal ideations and behaviors (29). The known association between low educational attainment and poverty in older adults may also reflect the low employment level of the older adults in our study (30–32). The prevalence of medical diseases is three times higher in the older group than in the younger group. In previous studies, medical illnesses such as cancer, liver disease, chronic obstructive lung disease, urinary problems, arthritis/arthrosis, moderate to severe pain, and neurologic and psychiatric diseases have been associated with suicidal behavior in the elderly (19, 33). Marital status and religion differed between the two groups. Although married attempters were predominant in both groups, older people were more likely to be separated (33.3%) while younger people were unmarried (40.1%), which is comparable with previous studies (34, 35). The older adults were more likely to be religious (45.5 vs. 25.4%) as per the recent report showing a trend toward decreased religiosity among young adults (36). This result is inconsistent with a previous study showing the protective effects of religion on suicide attempts (37) and may implicate the additional mechanism of SSA in old age. It also should be considered that differences in the demographic and lifestyle variables between older and younger suicide attempters might be general features of older and younger adult groups.

Information on suicide attempts differed between groups regarding precipitating factors, previous suicide attempts, and suicide completion rate. The major stressor regarding suicide attempt was physical illness in the older group (37.8%) and interpersonal problems in younger group (43.1%). Previous studies have also shown that the major reasons for suicide attempts included physical illness or problems in the older adults and quarrels with the spouse or family members in young adults (21, 35). Older people may face an inevitable vulnerability due to their poor physical health and lack of family or social support (28) that may burden them and lead them to commit suicide. The next major precipitating factors in the older group found in this study were interpersonal (21.6%) and economic problems (10.8%). Older people with suicidal behavior have chronic difficulties in interpersonal relationships and low overall social support (35). Underestimated depressive disorder or subclinical depressive symptoms are strong predictors and moderators of suicidal behavior (35, 38, 39). Thus, interpersonal problems and physical or medical problems should be considered while evaluating old individuals with a suicide attempt.

The older group had a 3-fold lower rate of previous suicide attempts (13.5 vs. 39.6%) and a 3-fold higher suicide completion rate (16.2 vs. 5.4%) than the younger group. Our study result corresponds with an earlier study that reported an ~3-fold suicide completion rate among the older attempters (16). Regarding the lethality of the suicide attempt, these results may imply that SSA is more prevalent in the older group than in the younger group. Increased lethality of suicide in old age has been consistently found in previous studies (15–17, 38, 40). The less-lethal suicidal behavior has also been predominantly found in adolescent and young adult groups (17, 20). Evidence shows that attempted and completed suicide exhibits opposite trends according to age (41). Although previous suicide attempt is a risk factor for suicide re-attempt and completion (9), it can be postulated that old individuals' vulnerability to such attempts and the high severity of their suicide attempts may be associated with a decreased number of previous suicide attempts in the present study.

Another important finding in this study is the difference in the factors related to lethal suicidal attempts between older and younger adult groups. Factors like old age, male sex, alcohol intoxication, presence of interpersonal stress, and history of schizophrenia were significantly associated with RRRS in the young adult group. However, no factors were associated with RRRS in the old adult group. Earlier studies have found several associated factors for completed or high-lethal suicide attempts in old age: depressive disorder (42), physical illness (42, 43), loss (43), and high suicidal intent (15, 44). Researchers have also reported that job problems, family discord, and alcoholism were less associated with completed suicide attempts among suicide attempters aged over 50 years (43). Since only a few studies have investigated the risk factor for high suicide lethality or SSA among old adults, little is known about the factors affecting the severity of attempted suicide, specifically in the old age group. In psychological aspects, characteristics of delaying small rewards and waiting for larger ones (45), vulnerable personality including high neuroticism and lower openness to experience are elevated in older suicide attempters with high-lethality (46). It was found that cluster B personality traits associated with repetitive, less severe suicide attempts decreased in old age (47) and older suicide attempters (48). The lack of personality trait information can contribute to the insignificant result in the older group in this study. Additionally, we assume that the small sample size and lack of information of possible risk factors such as cognitive decline, frailty, and social interaction (48–50) contribute to the results showing no significant risk factors for SSA in the old adult group. Since we analyzed the risk factors for high-risk suicide attempts, it may differ from those for suicide attempts in the old individuals. Besides, age was no longer associated with SSA after age 65, which may imply that age may be a predisposing factor for SSA in old age rather than a risk or precipitating factor (51). Cautious interpretation is needed due to the under-report or underestimation of the risk factors of older people. Although social factors such as bereavement and isolation were found to be predictive for suicide in old age (52), particular social factors such as Confucian thought, the rapid progress of modernization, and change to nuclear family in Korean society also should be considered. Regarding the lethality of suicide attempts itself in this study, RRRS was comparable between groups which might be attributed to the small sample size of the older group. Future studies with large number of old people is needed to find the differences in lethality and associated factors.

This study has several limitations. First, the main weakness is the relatively small sample size. Although there have been no large studies comparing older to younger suicide attempters, the sample size in the older group (*n* = 37) was too small to show enough statistical power. Future studies should try to include a comparable large number of older participants. Additionally, the sample was not based on the general population of suicide attempters but a single university hospital emergency room. Second, suicide attempters who refused the interview were not included in the study, and participants may have been underestimated because of the brief interview setting at the emergency department. To reduce this bias, we reviewed the charts of suicide attempters during the study period and included suicide victims in the study. Third, although we obtained comprehensive demographic and clinical data and information on suicidal behavior, we may have disregarded some other potential confounders, such as attempters' characteristics, socioeconomic status, and time of the suicide attempt which may be associated with a seasonal variation of the suicide attempt (53). Fourth, aging factors such as cognitive decline, frailty, and social interaction associated with suicide attempts were not collected (49, 50). Lastly, participants were all of Korean descent from a single tertiary hospital, limiting this study's generalizability to other populations or ethnic groups. Cultural, lifestyle, and ethnic differences could play a role in the risk factor for SSA in the old age group (54). Future studies should also consider the heterogeneity of the study participants, including recruiting sites and ethnicity.

## Conclusions

SSA in the old age is a major personal and social problem worldwide. We found that the usual suspects for SSA in younger suicide attempters, such as age, sex, interpersonal stress, and impression of schizophrenia, were not applicable in older suicide attempters. This suggests that a view different from the general population is needed to detect and prevent SSAs among the old people in the future. From 2011 to 2017, among OECD members, South Korea had the highest suicide rate among the older adults. In Korea, the “Act on Suicide Prevention and Respect for Life” was enacted in 2011. A suicide attempt follow-up service is provided in cooperation with the emergency and psychiatry departments in the emergency room (55, 56). The act also includes strategies for managing stress, establishing campaigns for life respect culture, improving media reporting of suicide, operating local suicide prevention centers for the old people (57). Except for a slight increase in 2015, the old age suicide rate is declining every year. Also, the gap between elderly suicide rates in South Korea and other OECD countries is gradually decreasing (58). Efforts should be made to find the modifiable and moderating risk factors through future studies that could contribute to public health surveillance strategies, education of the general population and professionals, and policy implementation for suicide prevention.

## Data Availability Statement

The datasets generated for this study are available on request from the corresponding author.

## Ethics Statement

The institutional review board of Gachon University Gil Medical Center approved this study. Because of a retrospective study design, the need for informed consent was waived (approval no. GCIRB2019-126).

## Author Contributions

DK: study concept and design, analysis and interpretation of data, and preparation of the manuscript. SC: study concept and design, interpretation of data, preparation of the manuscript, and funding acquisition. S-JC: study concept and design, acquisition of data, analysis and interpretation of data, preparation of the manuscript, and funding acquisition. SW, S-GK, and BY: study concept, interpretation of data, supervising and critical review, and editing the manuscript. All authors contributed to the article and approved the submitted version.

## Conflict of Interest

The authors declare that the research was conducted in the absence of any commercial or financial relationships that could be construed as a potential conflict of interest.

## References

[1] Naghavi M. Global, regional, and national burden of suicide mortality 1990 to 2016: systematic analysis for the global burden of disease study 2016. BMJ. (2019) 364:l94. 10.1136/bmj.l94 31339847 PMC6598639

[2] OECD. Health at a Glance 2019. Paris: OECD Publishing (2019).

[3] Shin H-Y Lee J-Y Song J Lee S Lee J Lim B Cause-of-death statistics in the republic of Korea, 2014. J Korean Med Assoc. (2016) 59:221–32. 10.5124/jkma.2016.59.3.221

[4] Gvion Y Levi-Belz Y. Serious suicide attempts: systematic review of psychological risk factors. Front Psychiatry. (2018) 9:56. 10.3389/fpsyt.2018.00056 29563886 PMC5845877

[5] Sun J Guo X Zhang J Wang M Jia C Xu A. Incidence and fatality of serious suicide attempts in a predominantly rural population in Shandong, China: a public health surveillance study. BMJ Open. (2015) 5:e006762. 10.1136/bmjopen-2014-006762 25673439 PMC4325129

[6] O'Dea D Tucker S The Cost of Suicide to Society. Wellington: Ministry of Health (2005).

[7] Beck AT Beck R Kovacs M. Classification of suicidal behaviors: I. Quantifying intent and medical lethality. Am J Psychiatry. (1975) 132:285–7. 10.1176/ajp.132.3.285 1115273

[8] Kim S Choi KH Lee KS Kim DJ Hong SC Lee HK. Risk factors for serious suicide attempts with high medical severity. Suicide Life Threatening Behav. (2019) 50:408–21. 10.1111/sltb.12597 31642549

[9] Bostwick JM Pabbati C Geske JR McKean AJ. Suicide attempt as a risk factor for completed suicide: even more lethal than we knew. Am J Psychiatry. (2016) 173:1094–100. 10.1176/appi.ajp.2016.15070854 27523496 PMC5510596

[10] Choo CC Harris KM Ho RC. Prediction of lethality in suicide attempts: gender matters. Omega. (2017) 80:87–103. 10.1177/0030222817725182 28828921

[11] Beautrais AL Joyce PR Mulder RT Fergusson DM Deavoll BJ Nightingale SK. Prevalence and comorbidity of mental disorders in persons making serious suicide attempts: a case-control study. Am J Psychiatry. (1996) 153:1009–14. 10.1176/ajp.153.8.1009 8678168

[12] Grunebaum MF Galfalvy HC Oquendo MA Burke AK Mann JJ. Melancholia and the probability and lethality of suicide attempts. Br J Psychiatry. (2004) 184:534–5. 10.1192/bjp.184.6.534 15172948

[13] Zalsman G Braun M Arendt M Grunebaum MF Sher L Burke AK. A comparison of the medical lethality of suicide attempts in bipolar and major depressive disorders. Bipolar Disord. (2006) 8:558–65. 10.1111/j.1399-5618.2006.00381.x 17042829

[14] Sher L Oquendo MA Richardson-Vejlgaard R Makhija NM Posner K Mann JJ. Effect of acute alcohol use on the lethality of suicide attempts in patients with mood disorders. J Psychiatric Res. (2009) 43:901–5. 10.1016/j.jpsychires.2009.01.005 19246050 PMC3767468

[15] Conwell Y Duberstein PR Cox C Herrmann J Forbes N Caine ED. Age differences in behaviors leading to completed suicide. Am J Geriatr Psychiatry. (1998) 6:122–6. 10.1097/00019442-199805000-00005 9581207

[16] McIntosh JL Santos JF Hubbard RW Overholser JC Elder suicide: research, theory and treatment: American psychological association. Geriatric Psychiatry. (1994) 16:827–8. 10.1037/10159-000

[17] Conwell Y Duberstein PR Caine ED. Risk factors for suicide in later life. Biol Psychiatry. (2002) 52:193–204. 10.1016/S0006-3223(02)01347-1 12182926

[18] Erlangsen A Stenager E Conwell Y. Physical diseases as predictors of suicide in older adults: a nationwide, register-based cohort study. Soc Psychiatry Psychiatric Epidemiol. (2015) 50:1427–39. 10.1007/s00127-015-1051-0 25835959

[19] Fässberg MM Cheung G Canetto SS Erlangsen A Lapierre S Lindner R. A systematic review of physical illness, functional disability, and suicidal behaviour among older adults. Aging Mental Health. (2016) 20:166–94. 10.1080/13607863.2015.1083945 26381843 PMC4720055

[20] Steele IH Thrower N Noroian P Saleh FM. Understanding suicide across the lifespan: a United States perspective of suicide risk factors, assessment & management. J Forensic Sci. (2018) 63:162–71. 10.1111/1556-4029.13519 28639299

[21] Suresh Kumar PN Anish PK George B. Risk factors for suicide in elderly in comparison to younger age groups. Indian J Psychiatry. (2015) 57:249–54. 10.4103/0019-5545.166614 26600577 PMC4623642

[22] Wertheimer J. Psychiatry of the elderly: a consensus statement. Int J Geriatric Psychiatry. (1997) 12:432–5. 10.1002/(sici)1099-1166(199704)12:4<432::aid-gps1576>3.0.co;2-s 9178046

[23] Blazer DG Depression in late life: review and commentary. Focus. (2009) 7:118–36. 10.1176/foc.7.1.foc118 12634292

[24] Blazer DG. Depression in late life: review and commentary. J Gerontol A. (2003) 58:M249–M65. 10.1093/gerona/58.3.M249 12634292

[25] Park JH Kim KW Kim MH Kim MD Kim BJ Kim SK. A nationwide survey on the prevalence and risk factors of late life depression in South Korea. J Affect Disord. (2012) 138:34–40. 10.1016/j.jad.2011.12.038 22284016

[26] First MB Gibbon M The structured clinical interview for DSM-IV axis I disorders (SCID-I) and the structured clinical interview for DSM-IV axis II disorders (SCID-II). In: Hilsenroth MJ, Segal DL, editors. Comprehensive Handbook of Psychological Assessment, Vol. 2. Personality Assessment. Hoboken, NJ: John Wiley & Sons Inc (2004). p. 134–43.

[27] Weisman AD Worden JW. Risk-rescue rating in suicide assessment. Arch Gen Psychiatry. (1972) 26:553–60. 10.1001/archpsyc.1972.01750240065010 5027119

[28] Heffner H “A dragon risen from a shallow stream” the south korean middle class framed through narratives of loss, progress, and return. Alternatives. (2015) 40:31–45. 10.1177/0304375415581256

[29] Iemmi V Bantjes J Coast E Channer K Leone T McDaid D. Suicide and poverty in low-income and middle-income countries: a systematic review. Lancet Psychiatry. (2016) 3:774–83. 10.1016/S2215-0366(16)30066-9 27475770

[30] Muller A. Education, income inequality, and mortality: a multiple regression analysis. BMJ. (2002) 324:23. 10.1136/bmj.324.7328.23 11777800 PMC61654

[31] OECD Education at a Glance 2018: OECD Indicators. Paris: OECD Publishing (2016).

[32] Yoon H-S. Korea: balancing economic growth and social protection for older adults. Gerontologist. (2013) 53:361–8. 10.1093/geront/gnt018 23528291

[33] Juurlink DN Herrmann N Szalai JP Kopp A Redelmeier DA. Medical illness and the risk of suicide in the elderly. JAMA Intern Med. (2004) 164:1179–84. 10.1001/archinte.164.11.1179 15197042

[34] Kim YR Choi KH Oh Y Lee HK Kweon YS Lee CT. Elderly suicide attempters by self-poisoning in Korea. Int Psychogeriatr. (2011) 23:979–85. 10.1017/S1041610211000263 21356160

[35] Szanto K Gildengers A Mulsant BH Brown G Alexopoulos GS Reynolds CF 3rd. Identification of suicidal ideation and prevention of suicidal behaviour in the elderly. Drugs Aging. (2002) 19:11–24. 10.2165/00002512-200219010-00002 11929324

[36] Kim J Lee Y Son J Smith TW Trends of religious identification in Korea: changes and continuities. J Sci Study Religion. (2009) 48:789–93. 10.1111/j.1468-5906.2009.01480.x

[37] Lawrence RE Oquendo MA Stanley B. Religion and suicide risk: a systematic review. Arch Suicide Res. (2016) 20:1–21. 10.1080/13811118.2015.1004494 26192968 PMC7310534

[38] O'Connell H Chin AV Cunningham C Lawlor BA. Recent developments: suicide in older people. BMJ. (2004) 329:895–9. 10.1136/bmj.329.7471.895 15485967 PMC523116

[39] Ross RK Bernstein L Trent L Henderson BE Paganini-Hill A. A prospective study of risk factors for traumatic deaths in a retirement community. Prev Med. (1990) 19:323–34. 10.1016/0091-7435(90)90032-F 2377594

[40] Conwell Y Thompson C. Suicidal behavior in elders. Psychiatr Clin North Am. (2008) 31:333–56. 10.1016/j.psc.2008.01.004 18439452 PMC2735830

[41] McIntosh JL Suicide: guidelines for assessment, management, and treatment. In: Bongar B, editor. Suicide of the Elderly. New York, NY: Oxford University Press (1992), 109–112.

[42] Conwell Y Lyness JM Duberstein P Cox C Seidlitz L DiGiorgio A. Completed suicide among older patients in primary care practices: a controlled study. J Am Geriatr Soc. (2000) 48:23–9. 10.1111/j.1532-5415.2000.tb03024.x 10642017

[43] Conwell Y Rotenberg M Caine ED. Completed suicide at age 50 and over. J Am Geriatr Soc. (1990) 38:640–4. 10.1111/j.1532-5415.1990.tb01422.x 2358625

[44] Kim H Ahn JS Kim H Cha YS Lee J Kim MH. Sociodemographic and clinical characteristics of old-old suicide attempters compared with young-old and middle-aged attempters. Int J Geriatr Psychiatry. (2018) 33:1717–26. 10.1002/gps.4976 30264415

[45] Dombrovski AY Szanto K Siegle GJ Wallace ML Forman SD Sahakian B. Lethal forethought: delayed reward discounting differentiates high-and low-lethality suicide attempts in old age. Biol Psychiatry. (2011) 70:138–44. 10.1016/j.biopsych.2010.12.025 21329911 PMC3125431

[46] Duberstein PR Conwell Y Caine ED. Age differences in the personality characteristics of suicide completers: preliminary findings from a psychological autopsy study. Psychiatry. (1994) 57:213–24. 10.1080/00332747.1994.11024686 7800770

[47] Lautenschlager NT Förstl H. Personality change in old age. Curr Opin Psychiatry. (2007) 20:62–6. 10.1097/YCO.0b013e3280113d09 17143085

[48] Szücs A Szanto K Wright AG Dombrovski AY. Personality of late-and early-onset elderly suicide attempters. Int J Geriatr Psychiatry. (2020) 35:384–95. 10.1002/gps.5254 31894591 PMC7291767

[49] Badre D. Cognitive control, hierarchy, and the rostro-caudal organization of the frontal lobes. Trends Cogn Sci. (2008) 12:193–200. 10.1016/j.tics.2008.02.004 18403252

[50] Lopez-Castroman J Moulahi B Azé J Bringay S Deninotti J Guillaume S. Mining social networks to improve suicide prevention: a scoping review. J Neurosci Res. (2020) 98:616–25. 10.1002/jnr.24404 30809836

[51] Lee H Seol KH Kim JW. Age and sex-related differences in risk factors for elderly suicide: differentiating between suicide ideation and attempts. Int J Geriatr Psychiatry. (2018) 33:e300–6. 10.1002/gps.4794 28967671

[52] Rubenowitz E Waern M Wilhelmson K Allebeck P. Life events and psychosocial factors in elderly suicides–a case–control study. Psychol Med. (2001) 31:1193–202. 10.1017/S0033291701004457 11681545

[53] Aguglia A Serafini G Solano P Giacomini G Conigliaro C Salvi V. The role of seasonality and photoperiod on the lethality of suicide attempts: a case-control study. J Affect Disord. (2019) 246:895–901. 10.1016/j.jad.2018.12.094 30795496

[54] Coleman KJ Johnson E Ahmedani BK Beck A Rossom RC Shortreed SM. Predicting suicide attempts for racial and ethnic groups of patients during routine clinical care. Suicide Life Threatening Behav. (2019) 49:724–34. 10.1111/sltb.12454 29574965 PMC6153081

[55] National Suicide Prevention Action Plan Sejong: Ministry of Health and Welfare Available online at: http://www.mohw.go.kr/react/al/sal0301vw.jsp?PAR_MENU_ID=04&MENU_ID=0403&page=44&CONT_SEQ=343649

[56] Park SC Na KS Kwon SJ Kim M Kim HJ Baik M “Suicide CARE” (standardized suicide prevention program for gatekeeper intervention in korea): an update. Psychiatry Invest. (2020) 17:911 10.30773/pi.2020.0166 PMC7538250 32933238

[57] Statutes of the Republic of Korea Act on the Prevention of Suicide and the Creation of Culture of Respect for Life. (2011). Available online at: https://elaw.klri.re.kr/eng_service/lawView.do?hseq=49889&lang=ENG (accessed August 1, 2020).

[58] Lee SU Park JI Lee S Oh IH Choi JM Oh CM. Changing trends in suicide rates in South Korea from 1993 to 2016: a descriptive study. BMJ Open. (2018) 8:e023144. 10.1136/bmjopen-2018-023144 30269071 PMC6169778

