# Supplementary material for: Risk Factors for Serious Suicide Attempts: Difference Between Older and Younger Attempters in the Emergency Department
Source: Front Psychiatry. 2021 Jan 8;11:607811. doi: 10.3389/fpsyt.2020.607811 (PMC7820120; doi:10.3389/fpsyt.2020.607811)
Supplement: Supplementary file 1 [file Data_Sheet_1.PDF]

Figure S1. Post hoc analysis of power achieved in the regression analysis performed in the elderly group.

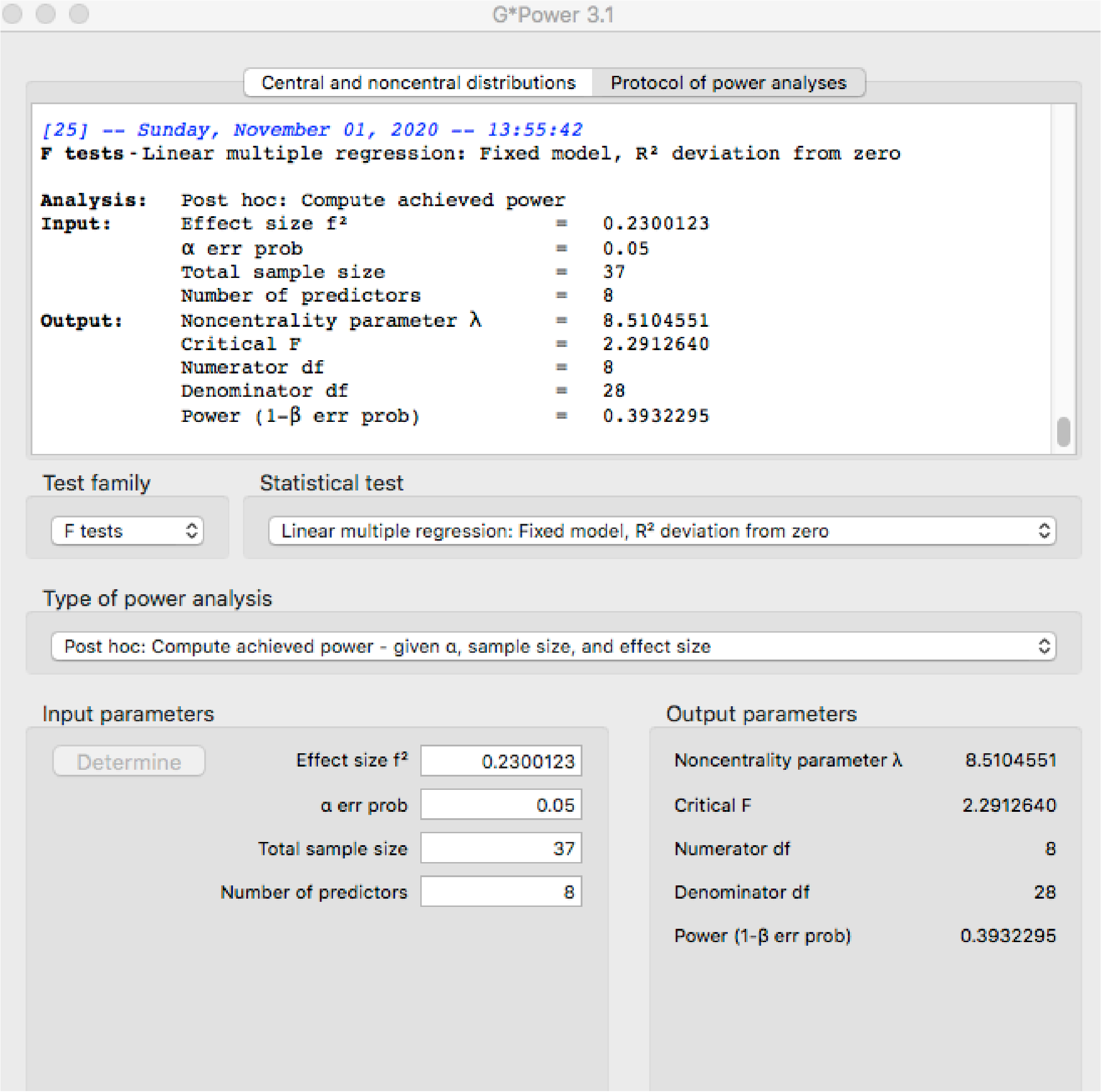

We calculated the post-hoc power achieved in this analysis using G*Power 3.1. The result is as follo w. For calculation of effect size F^2, we transformed effect size R^2 (0.187), which was yielded in the multiple regression analysis (enter method) in old age group (n=37), to f^2 in the G*Power program.
